# Insertion of short L1 sequences generates inter-strain histone acetylation differences in the mouse

**DOI:** 10.1186/s13100-024-00321-0

**Published:** 2024-05-10

**Authors:** Beverly Ann G. Boyboy, Kenji Ichiyanagi

**Affiliations:** Laboratory of Genome and Epigenome Dynamics, Department of Animal Sciences, Graduate School of Bioagricultural Sciences, Nagoya University, Furo-cho, Chikusa-ku, Nagoya, 464-8601 Japan

**Keywords:** LINE-1, Histone acetylation, Gene expression, KRAB-zinc finger proteins, Evolution

## Abstract

**Background:**

Gene expression divergence between populations and between individuals can emerge from genetic variations within the genes and/or in the *cis* regulatory elements. Since epigenetic modifications regulate gene expression, it is conceivable that epigenetic variations in *cis* regulatory elements can also be a source of gene expression divergence.

**Results:**

In this study, we compared histone acetylation (namely, H3K9ac) profiles in two mouse strains of different subspecies origin, C57BL/6 J (B6) and MSM/Ms (MSM), as well as their F1 hybrids. This identified 319 regions of strain-specific acetylation, about half of which were observed between the alleles of F1 hybrids. While the allele-specific presence of the interferon regulatory factor 3 (IRF3) binding sequence was associated with allele-specific histone acetylation, we also revealed that B6-specific insertions of a short 3′ fragment of LINE-1 (L1) retrotransposon occur within or proximal to MSM-specific acetylated regions. Furthermore, even in hyperacetylated domains, flanking regions of non-polymorphic 3′ L1 fragments were hypoacetylated, suggesting a general activity of the 3′ L1 fragment to induce hypoacetylation. Indeed, we confirmed the binding of the 3′ region of L1 by three Krüppel-associated box domain-containing zinc finger proteins (KZFPs), which interact with histone deacetylases. These results suggest that even a short insertion of L1 would be excluded from gene- and acetylation-rich regions by natural selection. Finally, mRNA-seq analysis for F1 hybrids was carried out, which disclosed a link between allele-specific promoter/enhancer acetylation and gene expression.

**Conclusions:**

This study disclosed a number of genetic changes that have changed the histone acetylation levels during the evolution of mouse subspecies, a part of which is associated with gene expression changes. Insertions of even a very short L1 fragment can decrease the acetylation level in their neighboring regions and thereby have been counter-selected in gene-rich regions, which may explain a long-standing mystery of discrete genomic distribution of LINEs and SINEs.

**Supplementary Information:**

The online version contains supplementary material available at 10.1186/s13100-024-00321-0.

## Introduction

Phenotypic variations between and within species can be introduced during evolution by gene expression changes. Such expression differences are due both to *cis* and *trans* effects. While *trans* effects involve the difference in expression level or function of proteins that regulate the expression of the gene of interest transcriptionally or post-transcriptionally, *cis* effects involve DNA sequence changes in linked regions involved in transcriptional or post-transcriptional regulation of gene expression. Epigenetics is a mechanism to regulate the transcriptional status of genes [1]. Epigenetic regulation includes chemical modification of chromatin components, such as methylation of DNA and acetylation and methylation of histones in promoter and enhancer regions. In general, promoter DNA methylation represses genes, and di- and tri-methylation of histone H3 at lysine-9 (H3K9me2 and H3K9me3) as well as H3K27me3 also represses genes. On the other hand, acetylation of various lysine residues of various histones, K9 and K27 of H3 (H3K9ac and H3K27ac) for example, enhances gene expression. These modifications alter the gene expression status of a given cell without a mutation in the underlying DNA sequence. Thus, it is likely that variations in the epigenetic states cause phenotypic variations. Of note is that, when comparing different species, species-specific epigenetic states are often associated with inter-specific differences in the genomic sequences, especially within transcription-factor binding sites [2–4].

During mouse evolution, various genetic changes have occurred and have been inherited in different mouse strains. The Mouse Genomes Project (MGP), which sequenced more than 50 closely related inbred mouse strains, has shown that there are strain-specific variations for disease genotypes and phenotypes such as immune response, diabetes, and cancer development [5, 6]. The changes in gene expression patterns can lead to phenotypic variation and be attributed to single nucleotide polymorphisms (SNPs), insertions-deletions (indels), and structural variations (SV) present in protein-coding regions, intergenic regions, and 5′ and 3′ untranslated regions (UTRs), which modify the landscape of *cis* regulatory elements (e.g., promoters, enhancers, silencers). A substantial amount of gene expression divergence has been identified between two mouse strains, C57BL/6 J and CAST/EiJ derived from mouse subspecies *Mus musculus domesticus* and *Mus musculus castaneus*, respectively [7, 8]. These differentially expressed genes were involved in circadian rhythm, glucose and fat nutritional state, infection/injury, chemical stimuli, and external stimuli. On the other hand, MSM/Ms (MSM) is derived from *Mus musculus molossinus*, which arose from the hybridization of *Mus musculus castaneus* and *Mus musculus musculus* in East Asia [9]. The divergence of *Mus musculus domesticus* and *Mus musculus molossinus* is estimated about 1 million years ago [9], and they show differences in various traits that are due to genetic differences with *cis* and *trans* effects [10–13]. There are ~ 15 million SNPs between B6 and MSM (a SNP density of one per ~ 160 bp, or ~ 6 SNPs in 1 kb) [14].

In addition to base substitutions, retrotransposition of long interspersed elements (LINEs), short interspersed elements (SINEs), and long-terminal-repeat elements (LTRs), are also important events that generate genetic and phenotypic variations in the mouse [15, 16]. Their retrotransposition not only disrupts genes [17], but also affects the expression of neighboring genes since transcription-factor binding sites are often carried, or have emerged, in their sequences [18–20]. In addition, these elements can affect epigenetic regulation. For example, polymorphic retrotransposon insertions are a source of epigenetic variation in primates [2, 3, 21]. In the mouse, B2 SINE copies inserted in B6 but not MSM have been shown to create a boundary of epigenetic modifications and generate gene expression differences between the strains [22]. Moreover, some resident retrotransposon copies (not insertional polymorphic) are variably DNA-methylated between individuals, generating metastable epialleles in a genetically identical population [23, 24]. Regarding global regulation of the epigenome, it has been proposed that the genomic distribution of retrotransposons partly dictates the higher order chromatin organization in the nucleus [25–27].

Among retrotransposons, L1 is the most abundantly populated in the mouse genome [5]. Full-length mouse L1, which is typically ~ 6 kb long, consists of two non-overlapping open reading frames (ORF1 and ORF2), a 5′ UTR that harbors an internal promoter, and a 3′ UTR with a poly A tail. Evolutionally young L1 copies are grouped into L1Md_A, L1Md_Tf, L1Md_Gf, L1Md_F, and L1Md_V, with L1Md_A and L1Md_Tf being youngest and retrotranspositionally most active currently. L1 copies are enriched in repressive B compartments and lamina-associated domains (LAD) in the nucleus [25, 28], and are depleted in gene promoter regions [29]. However, it was recently reported that polymorphic insertions of full-length L1Md_Tf copies help open chromatin formation in their neighboring regions in mouse embryonic stem cells, likely due to some transcription-factor binding sites present in the 5′ region [15].

In this study, the genetic mechanisms underlying histone acetylation differences between B6 and MSM were investigated. Firstly, published ChIP-Seq data of H3K9ac for the pure strains and their reciprocal F1 hybrids were analyzed for the presence of strain-specific and allele-specific H3K9ac regions, respectively. Secondly, mRNA-seq analysis of the F1 hybrids was carried out, which revealed a link between allele-specific histone acetylation and gene expression. Genetic mutations (SNPs) bearing or disrupting binding sites of the IRF3 transcription factor were associated with the histone acetylation difference. We also revealed that polymorphic insertions of even very short L1 copies were associated with the downregulation of acetylation. These results suggest an evolutional selection where L1 insertions in gene-rich regions are disfavored.

## Results

### Strain-specific histone H3 acetylation peaks were identified in the B6 and MSM strains

**Fig. 1:**
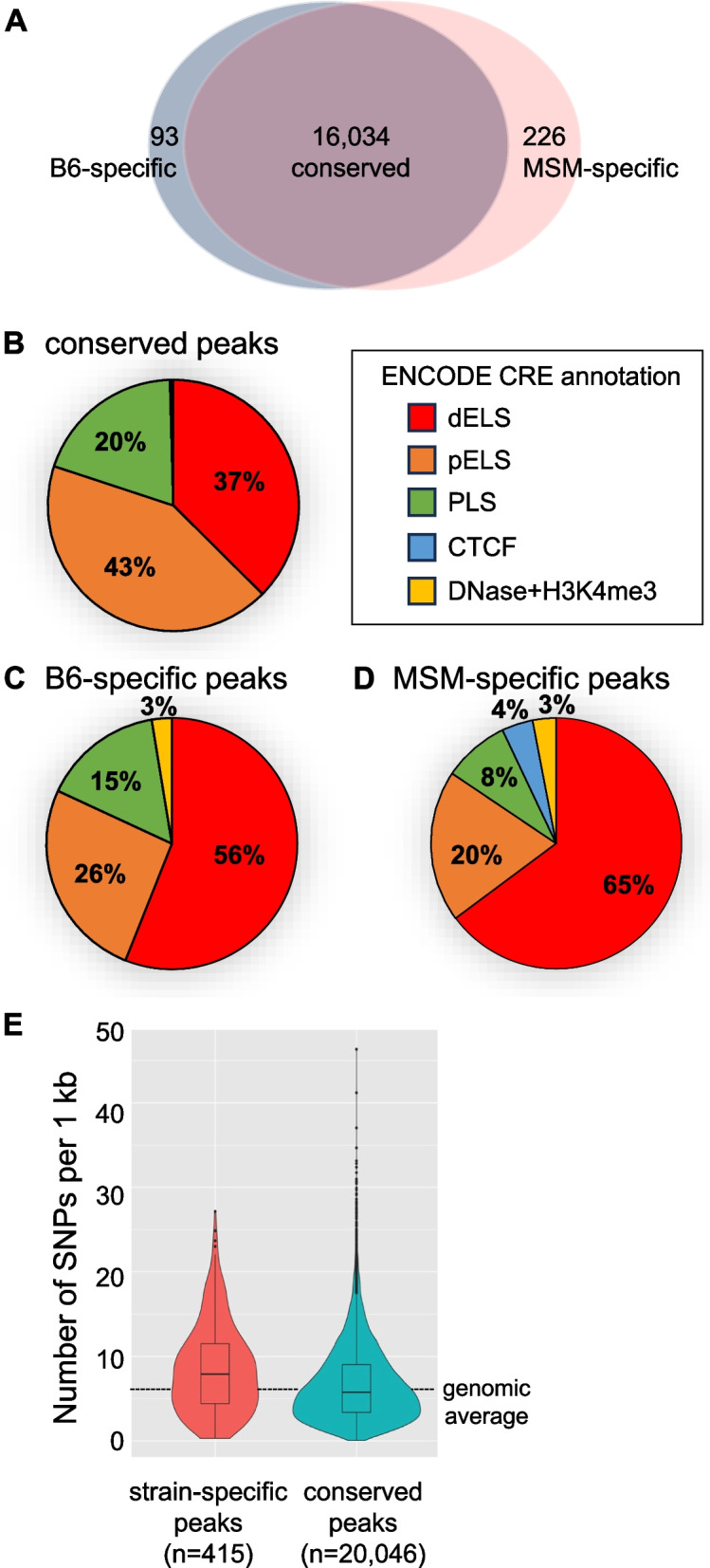
Identification and characterization of strain-specific H3K9ac peaks. **A** Venn diagram for shared, B6-specific, and MSM-specific peaks identified in this study. **B-D** Fraction of annotated *cis* regulatory elements (CREs) within conserved (B), B6-specific (C), or MSM-specific (D) peaks. dELS, distal enhancer-like sequence; pELS, proximal enhancer-like sequence; PLS, promoter-like sequence; DNase+H3K4me3, DNase hypersensitivity plus H3K4me3; CTCF, CTCF binding sites. **E** Statistics of SNP density in strain-specific and non-strain-specific peaks. The genome-averaged SNP density is shown as a dashed line. Details are available in supplementary table S1

Since H3 acetylation is deposited in regulatory regions to activate gene expression, the H3K9ac ChIP-Seq data of two inbred mouse strains, B6 and MSM, were obtained in our previous study [22]. To compare them, we investigated the data by mapping the ChIP-seq reads onto the B6 reference genome with all SNP positions being masked as N [14] using Hisat2 followed by identifying peaks using MACS2. This yielded a total of 16,353 peaks (identified in both or either strain). Then, to remove any mapping bias, we counted the numbers of input and ChIP reads mapped in each peak and calculated the fold enrichment of ChIP reads over the input. Whereas most of the peaks showed high degrees of ChIP enrichment in both strains, 93 (0.6%) and 226 (1.4%) peaks were specific to B6 and MSM, respectively (ChIP fold enrichments were > 2.5 in one strain and < 1.5 in the other strain) (Fig. 1A, supplementary table S1, Supplementary Material online). Most of the H3K9ac peaks resided in the candidate regulatory elements (CREs) [30], suggesting their involvement in gene regulation. A significant number – 14,331 (89%) of the 16,034 conserved peaks, contained annotated CREs (*p* < 0.001 by random permutation test) such as distal enhancers, proximal enhancers, and promoters (Fig. 1B). About two-thirds of B6- and MSM-specific peaks also contained annotated CREs (*p* < 0.001 by random permutation test; Fig. 1C and D). For these strain-specific peaks, distal enhancers (dELS) were more enriched (56 and 65%) in comparison to the conserved peaks (37%) (*p* < 10^−20^ by χ^2^ tests), suggesting a flexible activity of distal enhancers during evolution.

It is possible that the difference in the H3K9ac level resulted from sequence differences in these regions as well as differences in the expression and/or function of *trans*-acting factor(s) between the strains. Therefore, we compared the densities of SNPs in conserved and strain-specific peaks (Fig. 1E), revealing that SNP density was slightly higher in the strain-specific peaks, but it was not statistically significant (medians were 5.8 and 7.9 for conserved and strain-specific peaks, respectively, and the genomic average was 6.3). This implies that these differentially acetylated regions (DARs) were not evolutionally accelerated regions (showing higher local mutation rates). These results hint us that most of the acetylation differences are due to trans-acting factor(s) or that only a minor proportion of SNPs have a significant impact on H3K9ac in the liver.

### Allele-specific H3K9 acetylation peaks were detected in F1 hybrids between B6 and MSM

**Fig. 2:**
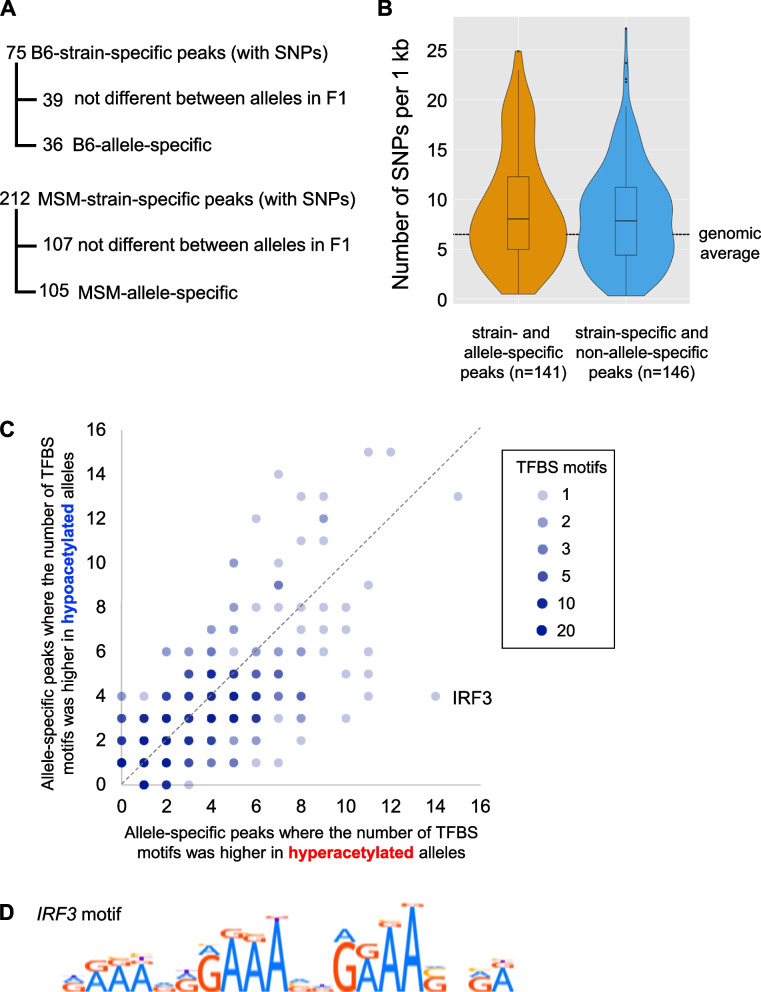
Identification and characterization of allele-specific H3K9ac peaks. **A** Number of allele-specific peaks identified in this study. Details are available in supplementary table S2. **B** Statistics of SNP density in allele-specific and non-allele-specific peaks. C Scatter plot of the allele-specific peaks in which the number of TFBS motifs was *higher* in hyperacetylated alleles than hypoacetylated alleles (x-axis) and those wherein the number of TFBS motifs was *lower* in hyperacetylated alleles than hypoacetylated alleles (y-axis). **D** Sequence logo representation of the IRF3 binding sequence motif

To study whether the DARs are made by *cis* or *trans* effects, we analyzed the H3K9ac ChIP-Seq data for F1 hybrids of B6 and MSM that were reciprocally crossed (B6/MSM and MSM/B6 of mother/father pair). For this analysis, we again used the SNP-masked reference genome for read mapping, and the allelic origin of the reads was identified based on the SNP data. Because at least one SNP must exist in a peak to discriminate the alleles, 18 B6-specific and 14 MSM-specific peaks were excluded from analysis due to the absence of a SNP. Thus, we analyzed 75 B6-specific and 212 MSM-specific peaks. To obtain the allele frequencies, the data from reciprocal F1 hybrids were averaged. Out of the 75 B6-specific and 212 MSM-specific peaks, 36 (48%) and 105 (50%) peaks, respectively, showed allele-biased mapping of ChIP reads (B6-allele frequencies of ChIP reads were > 0.68 for B6-biased, and < 0.32 for MSM-biased) in both reciprocal hybrids in a way consistent with the acetylation difference between the pure strains (Fig. 2A and supplementary table S2, Supplementary Material online). For these peaks, the acetylation difference should be made by a *cis* effect(s). However, these allele-biased peaks had a similar number of SNPs to those in non-allelic peaks (Fig. 2B), suggesting that the vast majority of the sequence changes in these regions are neutral in terms of H3K9ac in the liver. However, it is possible that a small number of sequence changes occurred in binding sites of transcription factors (TFs) that stimulate histone acetylation. Therefore, using Find Individual Motif Occurrences (FIMO) of the MEME suite program [31], we searched TF-binding motifs that are present only in the acetylated or non-acetylated alleles. Whereas allele-specific presence was not correlated to the state of acetylation for most TF-binding motifs, the binding motif for interferon regulatory factor 3 (IRF3) was significantly associated with acetylated alleles (Fig. 2C and D): it was present in 14 hyperacetylated alleles (10% of the all allele-specific peaks) but absent in their hypoacetylated counterpart, whereas it was present in 4 hypoacetylated alleles but absent in their hyperacetylated counterpart (*p* < 0.05 by χ^2^ test). By comparing to the genomic sequence of *Mus spretus* [32], the ancestral sequences of these peaks were inferred, revealing that there were 9 gain events of the IRF3 motif in hyperacetylated alleles and 5 loss events in hypoacetylated alleles. IRF3 is known to activate the expression of its target genes in many types of cells [33], suggesting that an evolutionary gain and loss of this binding motif are involved in the gain and loss of histone acetylation, respectively.

### Short L1 3′ UTR insertions induced hypoacetylation

**Fig. 3:**
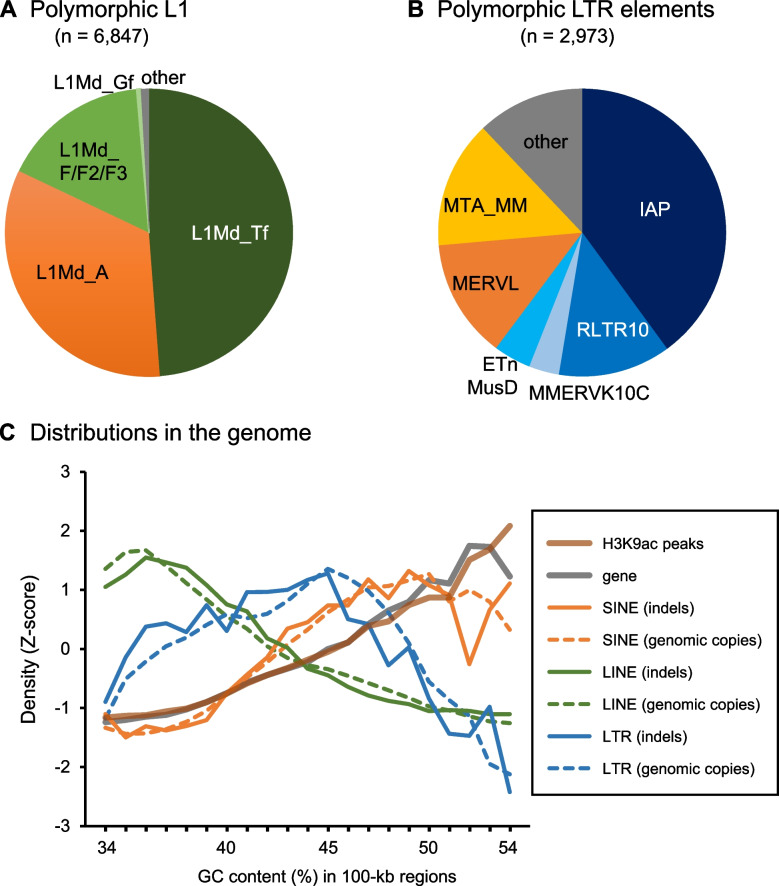
Identification and characterization of insertionally polymorphic retrotransposon copies. **A** Subfamilies of L1 indels identified in this study. **B** Groups of LTR retrotransposon indels identified in this study. Details are available in supplementary tables S3 (LINEs) and S4 (LTRs). **C** Distribution of H3K9ac peaks, genes, indels, and genomic copies of retrotransposons in the mouse genome with regard to regional GC contents

Gene expression differences and epigenetic differences in their regulatory sequences can also arise from structural variation of the genome, such as insertional polymorphism of retrotransposons. For example, the insertion of B2 SINE modifies DNA methylation, histone acetylation, and gene expression at the site of insertion in the mouse liver [22], and the insertion of full-length L1 LINE induces open chromatin in the mouse embryonic stem cells whereas the insertion of IAP retrotransposon (which is LTR-type) induces heterochromatin [15]. To study the impact of strain-specific insertion of retrotransposons, using the genomic sequence data [14], we have identified insertional polymorphism between the two strains. In addition to the ~ 1700 SINE insertion polymorphism we previously reported in our SINE study [22], herein we identified polymorphic insertions of 6847 LINEs and 2973 LTRs (Fig. 3 and supplementary tables S3 and S4, Supplementary Material online). In this analysis, we selected indels whose whole sequences were determined. Thus, most (6800 LINE insertions and all LTR insertions) were B6-specific insertions because whole sequences of long insertion in the MSM genome cannot be determined using Sanger sequencing reads. The polymorphic LINE insertions contained 3317 L1Md_Tf (49%), 2262 L1Md_A (33%), 1127 L1Md_F/F2/F3 (17%), and 31 L1Md_Gf (0.5%) insertions. The polymorphic LTR-element insertions contained 1186 IAP (40%), 376 RLTR10 (13%), 101 MMERVK10C (3.4%), 28 ETnERV (0.9%), 398 MERVL/MT2 (13%), and 423 MTA_MM (14%) insertions. To characterize their insertion-site preference, we divided the genome sequence into 100-kb regions and counted the LINE, LTR, and SINE indels as well as genes, acetylation peaks identified in the liver of the B6 pure strain, and non-polymorphic LINEs, LTRs, and SINEs in each region. These regions were then sorted by the GC content (Fig. 3C). This revealed that LINE indels have been retained in acetylation- and gene-poor regions, whereas SINE indels have been retained in acetylation- and gene-rich regions despite that their insertion sites are determined by the same enzyme, the LINE-encoded reverse transcriptase/endonuclease.

**Fig. 4:**
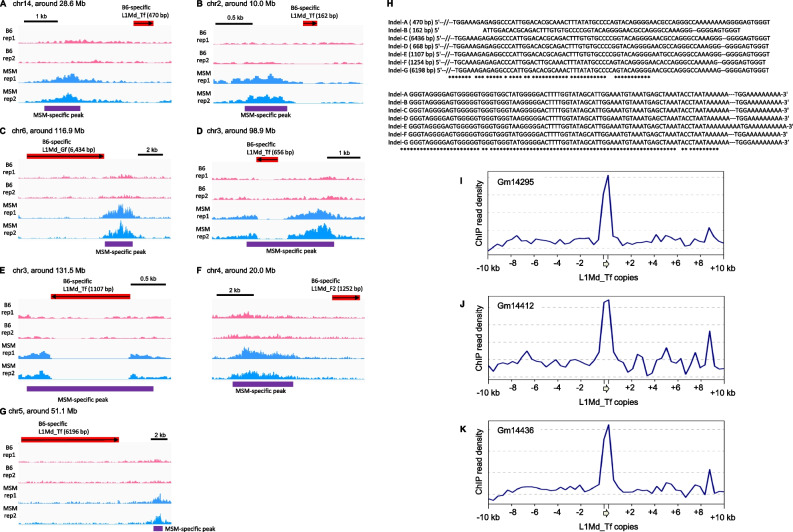
B6-specific L1 insertions associated with MSM-specific H3K9ac peaks. **A–G** IGV snapshots around L1 insertions. ChIP read densities are shown in pink (B6, two replicates) and light blue (MSM, two replicates). H3K9ac peaks are shown below in purple. Positions of Mb-specific L1 insertions are shown in red. Arrow indicates the direction of L1 and their length is shown in parentheses. **H** Alignment of the 7 inserted L1 sequences. Only the 3′ region is shown. Numbers in parentheses indicate the lengths of inserted copies. **I–K** Average ChIP read density of mouse KZFPs, Gm14295 (I), Gm14412 (J), and Gm14436 (K) in regions neighboring short L1Md_Tf copies in mm10 (100–400 bp containing the 3′ end, *n* = 2424). The ChIP data analyzed for mouse embryonic stem cells were obtained from GEO (accession number, GSE115291). The plots were generated using deepTools

We then compared DARs and the polymorphic sites of LINEs, SINEs, and LTRs. Whereas none of the B6-specific DARs was associated with MSM-specific insertions presumably due to the small number of the insertions, we identified 7 (3.1%) MSM-specific DARs that were closely located to B6-specific insertions (Fig. 4) (*p* = 0.057 by random permutation test). In all cases, the insertion of L1 (5 L1Md_Tf, 1 L1Md_Gf with 3′ region of L1Md_Tf, and 1 L1Md_F2 insertions) was associated with a complete loss of acetylation in its neighboring region, although the vast majority (94%) of strain-specific L1 insertions were located in hypoacetylated regions (see Fig. 3C). It should be noted that 6 of the 7 DARs had no SNP within them, strongly suggesting that the L1 insertions were the main cause. We also note that all of the 7 insertions contained the highly conserved 3′ UTR sequences, although the insertion lengths were variable from 162 bp to 6.4 kb (Fig. 4H). These results suggest that the insertion of a 3′ fragment of L1 reduced histone acetylation in the neighboring region. Krüppel-associated box domain-containing zinc finger proteins (KZFPs) work to make repressive chromatin in retrotransposons and genes [34]. They form a complex with Trim28, the Setdb1 H3K9 methylase, and histone deacetylases [35], resulting in histone deacetylation in their target genomic regions. It has been reported that four KZFPs, namely Gm14406, Gm14295, Gm14412, and Gm14436, preferentially bind to L1Md_Tf sequences [36]. Mapping of the published ChIP-seq reads for these KZFPs onto the mm10 reference sequence confirmed that except for Gm14406 (data not shown), the ChIP read density was high at and around L1Md_Tf copies of 100–400 bp in length (similar to the lengths of Indel-A, −B, and -D in Fig. 4H) which consisted of the 3′ region only (Fig. 4I–K).

**Fig. 5:**
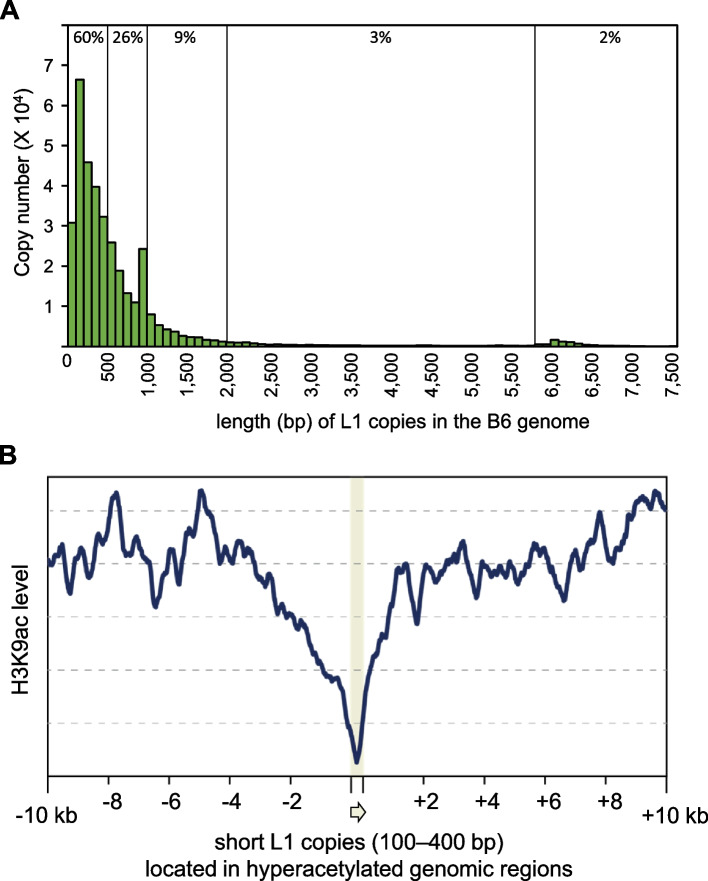
Genome-wide effects of L1 sequences on histone acetylation. **A** Length distribution of L1 copies present in the B6 genome (mm10). Only copies having the 3′ end were analyzed. The numbers above indicate the fractions of copies out of the total copies (60% for copies of < 0.4 kb, 26% for 0.4–1 kb, 9% for 1–2 kb, 3% for 2–5.8 kb, and 2% for > 5.8 kb (full-length or nearly full-length)) **(B)** Average H3K9ac level in the B6 strain of genomic regions near L1 copies. Copies resided in hyperacetylated regions and were less than 400 bp in length were used for the analysis (*n* = 845). The plot was generated using deepTools

L1 retrotransposition is carried out by a mechanism called target-primed reverse transcription, in which cDNA is generated from the 3′ polyA sequence of the RNA template toward the 5′ end [37, 38]. Premature termination of reverse transcription thus results in the insertion of a truncated L1 sequence consisting of only the 3′ region. Therefore, although the full-length L1 is about 6 kb in length, the vast majority of L1 insertions in the genome (not limited to L1Md_Tf copies) were much shorter than the full-length sequence (Fig. 5A, about 60% were < 400 bp). We underscore that not only the full-length L1 insertions but also short 3′ L1 fragments were enriched in repressive B compartments [28, 39] and gene-poor hypoacetylated regions (Fig. 3C), suggesting the involvement of these L1 fragments in gene repression. To see if even short 3′ L1 fragments can downregulate histone acetylation of neighboring regions, we selected genomic L1 copies (not necessarily an inter-strain indel), which were 100–400 bp in length, contained the 3′ end, and resided in hyperacetylated genomic regions (which showed regional fold enrichment of H3K9ac ChIP (B6) of ≥1.5 corresponding to 3% of 50-kb genomic regions). Then, we calculated histone acetylation levels in B6 around these L1 fragments. This revealed that, even in the hyperacetylated regions, the level of acetylation was dropped in regions within 2 kb from the insertion of the short L1 fragments (Fig. 5B), consistent with the L1 activity to downregulate acetylation in their neighbors. Moreover, analyses of published ChIP-seq data for other tissues disclosed similar pattern of downregulation of histone acetylation around the L1 fragments (supplementary Fig. 1).

### Allele-specific acetylation was associated with gene expression differences

**Fig. 6:**
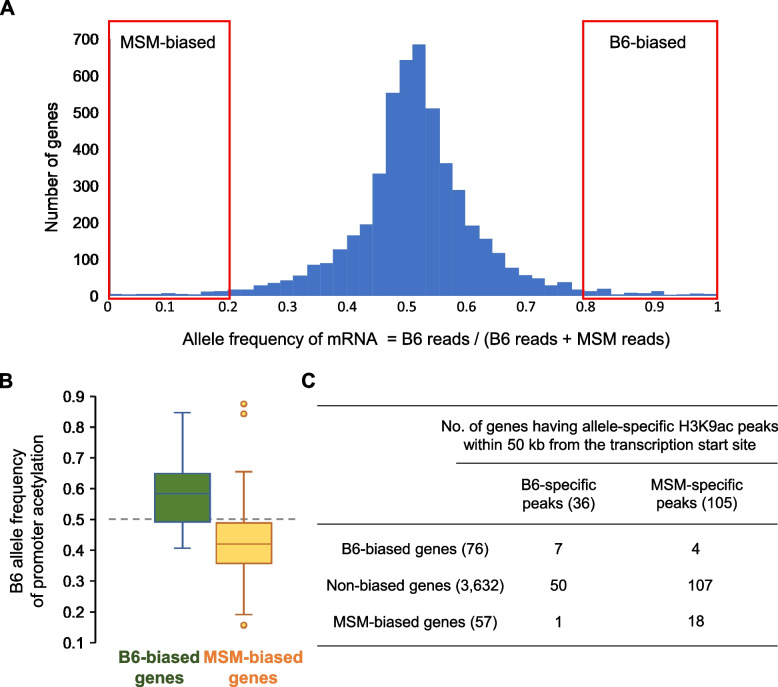
Allele-biased promoter acetylation is linked to allele-biased gene expression. **A** Statistics of allele frequency for genes expressed in liver of F1 hybrids. For each gene, the frequencies determined in the F1 progeny of reciprocal crosses were averaged. **B** Statistics of allele frequency (the average of reciprocal F1 hybrids) of H3K9ac ChIP reads mapped to promoters of genes showing B6- or MSM-biased expression. **C** Number of genes having allele-specific H3K9ac peak(s) within 50 kb among genes showing B6- or MSM-biased expression. Details are available in supplementary table S5

To study the relationship between the allelic biases of H3K9ac and gene expression, we performed mRNA-Seq for F1 hybrids. Similarly to allelic acetylation analysis, the B6-allele frequencies observed in reciprocal F1 hybrids were averaged. Out of 3632 genes whose allelic bias could be calculated, 76 genes had B6-allele-biased expression (B6-allele frequency of mRNA-seq reads ≥0.8, see Materials and Methods*)* while 57 genes were found to be MSM-biased (B6-allele frequency ≤ 0.2) (Fig. 6A and supplementary table S5, Supplementary Material online). Since it is thought that increased acetylation in promoter regions allows a higher level of transcription, we compared the allelic biases of promoter acetylation and expression of these genes (Fig. 6B). This revealed a general tendency that the allelic difference in promoter acetylation is positively correlated with the allelic difference in expression (*p* < 0.01 by Mann-Whitney U-test). In particular, promoters of 13 allele-biased genes (9.8% of a total of 133 genes) overlapped with allele-specific acetylation peaks identified above. These results suggest that the changes in promoter sequence result in changes in histone acetylation level, which in turn results in changes in transcription frequency. We note that the allelic bias of promoter acetylation was smaller than the allelic bias of gene expression, suggesting that other factors are also involved in the gene expression difference.

Because the majority of the allele-specific acetylation peaks we identified resided in enhancers (Fig. 1), we tested if they tended to reside in the vicinity (within 50 kb) of the genes showing biased expression. Although not so many genes, 7 B6- and 18 MSM-allele-biased genes had B6- and MSM-allele-specific acetylation peaks in the regions close to their transcription start sites, respectively (*p* < 0.01 by χ^2^ test) (Fig. 6C). These results suggest that genetic and epigenetic differences in enhancers underlie some, although not all, of the transcriptional differences that arose during mouse evolution.

## Discussion

Changes in gene expression are involved in the changes in phenotype in evolution. Such gene expression changes can arise by the alteration of epigenetic modifications, such as histone acetylation and methylation, in *cis* regulatory regions. This study aims to determine the genetic basis of changes in histone acetylation during evolution using two mouse strains derived from different subspecies, *Mus musculus domesticus* (B6) and *Mus musculus molossinus* (MSM), which diverged about 1 million years ago. These strains and their hybrids allowed us to analyze the *cis* and *trans* effects of epigenetic changes. Our results revealed that the vast majority of H3K9ac peaks were conserved between the strains and located in annotated *cis* regulatory elements such as promoters, which is consistent with that H3K9ac is frequently located around transcription start sites of active genes [40]. In addition, this mark was enriched in enhancers, together suggesting that H3K9ac is involved in gene regulation. It has been reported that about 30% of gene expression differences between mouse strains are explained by sequence changes [41]. Similarly, among the 319 strain-specific acetylation regions we identified here, 287 contained SNPs and 113 showed allelic differences in F1 hybrids. Therefore, 35% (113 out of 319) of inter-strain differences in histone acetylation can be explained by the differences in the underlying genetic sequences. The rest (strain-specific DARs but not showing allelic bias) may be caused by *trans* effects. Similar results were reported for inter-strain variance in open chromatin regions using various inbred strains [42]. Searching TF binding motifs in the allele-specific peaks identified here revealed that sequence changes in the motif for IRF3 are associated with acetylation changes — SNPs that disrupt or generate the IRF3 motif induce hypo- and hyper-acetylation, respectively. IRF3 is known to activate the expression of type I interferons as well as interferon-responsive genes upon viral infection [33]. We note that these cases were only a minority, and many allele-biased peaks lacked apparent association with SNPs generating or disrupting TF binding motifs. This is presumably because of a lack of knowledge of TFs decreasing histone acetylation --- our knowledge (i.e., the TF binding motif database) is biased toward TFs increasing histone acetylation. Therefore, it remains possible that differential binding of histone deacetylation-associated TFs accounts for differential acetylation between the alleles.

Our RNA-seq analysis of the reciprocal F1 hybrids showed that, out of 3632 genes expressed in the liver and was able to be analyzed for allele frequencies, 133 genes (3.7%) showed allele-biased expression. We showed that these genes tend to have allele-biased acetylation in their promoters. Moreover, we revealed that allele-specific acetylation peaks were preferentially located near the genes showing allele-biased expression, suggesting that epigenetic changes in enhancers alter the gene regulatory program. These findings reflected the previous results showing that, despite being reared in the same environment and sharing a last common ancestor less than a million years ago, subtle phenotypic differences exist between the inbred mouse strains, C57BL/6 J and CAST/EiJ, due to *cis-* and *trans-*acting variants that shape the gene regulatory network [8].

The most intriguing finding in this work are the effect of 3′ L1 fragments on histone acetylation. The 5′ region of L1 copies in the mouse genome provides binding sites for TFs such as HNF4α (older copies) and STAT1 (younger copies), and consequently, their insertional polymorphism results in inter-strain variability of open chromatin [42]. Although these suggest the activating roles of L1 insertions, the mouse L1 sequences are depleted in gene-rich A compartments, especially depleted in gene promoter regions [28, 29]. In contrast, B1 and B2 SINEs are enriched in the A compartments [28, 29, 39]. Such preferential localization is unlikely due to their insertion-site preference because L1 and the SINEs are retrotransposed by the same enzyme encoded in L1 [43]. Thus, their different preference in genomic localization remains a mystery. A possible explanation would be that L1 insertions in gene-rich regions have been counter-selected during evolution. Indeed, inter-strain indels of L1 were preferentially retained in gene-poor regions but not in gene-rich regions (Fig. 3C). This could be because they are marked with H3K9me3, a repressive chromatin modification, which then propagates to their neighborhood. However, H3K9me3 is generally accumulated only in the 5′ promoter region of L1 [44]. Most L1 sequences in the genome lack the 5′ promoter region because L1 retrotransposition predominantly generates incomplete insertions that have only a short sequence of the 3′ regions. This argues that the H3K9me3 mark at L1 is not the cause of the counter-selection. What could be the negative effect of short L1 insertions in the gene-rich regions? Our results revealed that insertions of even short L1 sequences in the genome downregulate histone acetylation of their neighboring regions, which may lower the expression of neighboring genes. Furthermore, our results suggest that the downregulation of acetylation is possibly mediated by KZFPs, especially, Gm14295, Gm14412, and Gm14436, that bind to regions of the L1 sequence including the 3′ region. KZFPs have been rapidly amplified in mammals to counteract the activity of newly emerging retrotransposons by inhibiting their transcription [34], thus inhibiting their retrotransposition. In addition to this accepted view, the data presented here argues in favor of a hypothesis that the KZFP-mediated silencing system also limits L1 amplification after retrotransposition by making such retrotransposed copies inserted close to genes be eliminated from the population of the host (i.e., selection against inserted copies).

We previously showed that B2 SINE also affects histone acetylation [22]. In the SINE case, B2 inhibits the expansion of H3K9ac regions around promoters. As a result, H3K9ac regions were shortened, which is regarded as having a negative effect on acetylation. However, we underscore that B2 does not disrupt H3K9ac regions. This is the point of difference between LINEs and SINEs in view of their effects on histone acetylation. The mechanistic difference between the two types of retrotransposons to affect chromatin modifications may underlie their clear differences in their genomic distributions.

## Materials and methods

### RNA extraction, RNA library preparation, and sequencing

All animal experiments were approved by the committee of Nagoya University and carried out according to the animal welfare guidelines.

For mRNA-seq analysis, the liver of 5-month-old female F1 hybrids (age-, sex-, and tissue-matched with the published H3K9ac ChIP-seq data) were used; 2 progenies of a B6 mother and an MSM father and 2 progenies of an MSM mother and a B6 father. Total RNAs were extracted from ~ 30 mg of homogenized tissues using the Directzol™ RNA MicroPrep Kit (ZymoResearch, Irvine, CA, USA) according to the manufacturer’s instructions. The samples were quality-checked using Qubit (Thermo Fisher Scientific, Waltham, MA, USA) and 2100 Bioanalyzer (Agilent Technologies, Santa Clara, CA, USA). Then, mRNA-Seq libraries were prepared using the NEBNext directional poly(A) mRNA library preparation kit (New England BioLabs, Ipswich, MA, USA). The libraries were sequenced in the 150-bp paired-end mode on HiSeq X Ten (Illumina, San Diego, CA, USA) by Macrogen Japan (Tokyo, Japan), yielding 56–88 million read pairs.

### Gene expression analysis

Trim Galore! (https://www.bioinformatics.babraham.ac.uk/projects/trim_galore/) was used to perform adapter trimming on the mRNA-Seq reads using the options, −q 30 --length 30 --paired --three_prime_clip_R1 1 three_prime_clip_R2 2. Read alignment was done using Tophat2 [45] with masked mm10 as the reference genome (wherein the nucleotides at SNP sites were changed to N), using alignment options, −G --library-type fr-firststrand -g 1. Next, SNPsplit [46] was used to discriminate the B6 and MSM alleles from the aligned reads based on known SNP positions. Then, using Cuffdiff [45] with the bam files for the B6- and MSM-derived reads, respectively, the number of mapped reads were counted for each gene (shown in the genes.read_group_tracking files generated by Cuffdiff) to calculate the allele frequencies (the number of B6-derived reads divided by the total of B6- and MSM-derived reads). Genes were regarded as B6-biased if the allele frequency was 0.8 or more, whereas genes were regarded as MSM-biased if the allele frequency was 0.2 or less.

### Analysis of published ChIP-Seq H3K9ac data

The H3K9ac ChIP-seq published data [22] from liver tissues of B6 and MSM pure strains, as well as F1 reciprocal hybrids (MSM[mat] × B6[pat] and B6[mat] × MSM[pat]), were used (GSM4728978 to GSM4728989). After trimming of adapter sequence and low-quality reads using Trim Galore!, the reads were mapped onto the SNP-masked mm10 reference genome using Bowtie2 [47]. Next, the H3K9ac broad peaks were determined using MACS2 [48] with the options, callpeak --broad --slocal 1000 -B -f BAMPE -g mm. The locations of the peaks obtained for B6 and MSM pure strains were then merged. Using the ‘coverage’ function of BEDTools [49], the ChIP and Input reads were counted for each peak.

To identify pure strain-specific peaks, the fold enrichment (FE = IP/Input) of all samples (two B6 and two MSM) was calculated. Then, B6-specific peaks were determined if the FE (B6) ≥ 2.5 and FE (MSM) ≤ 1.5, while MSM-specific peaks were determined if the FE (MSM) ≥ 2.5 and FE (B6) ≤ 1.5. Peaks were intersected with *cis* regulatory regions determined by ENCODE as promoter-like, proximal enhancer-like, distal enhancer-like, with CTCF binding, and with DNase-H3K4me3 binding regions.

To identify allele-specific peaks, the input and IP reads of F1 hybrids were mapped to the SNP-masked reference genome. After discriminating the alleles for each read by SNPSplit, reads mapped to the peaks identified in B6 and/or MSM pure strains were counted using BEDTools. Then, the allele bias (the number of B6-derived reads divided by the total of B6- and MSM-derived reads) was computed for each peak. Peaks were regarded as ‘B6-allele-biased’ if the allele bias was 0.68 or more, whereas peaks were regarded as ‘MSM-allele-biased’ if the allele bias was 0.32 or less. To identify potential transcription-factor binding sites in these allele-biased peaks, the B6 (mm10) and MSM (mm10 but SNP positions changed to the MSM sequence) sequences of these peaks were analyzed by FIMO [50] using the position weight matrix files obtained from the HOCOMOCO database [51].

### Identification of insertional polymorphic retrotransposons and analysis of their association with strain- and allele-specific H3K9ac peaks

To identify indels between B6 and MSM, each shotgun read generated in genome sequencing of MSM [14] was blasted against the mm10 reference genome with a minimum gap penalty. Short indels (< 300 bp) were identified in alignments with gaps (in either B6 or MSM), which yields many SINE indels [22]. To obtain long indels, reads were collected if the 5′ part was matched to a part of the mm10 genome and the rest was matched to a distant part of mm10 in the same chromosome and same orientation. The regions sandwiched by the two matched regions were regarded as insertions in B6 (deletion in MSM). These indels were then manually analyzed on the UCSC Genome Browser [52], yielding indels of LINE and LTR elements. Of the indels identified, randomly selected 24 loci were confirmed by PCR. Note that long MSM-specific insertions cannot be identified due to the lengths of sequencing reads (~ 700 bp or shorter).

### Analysis of KZFP binding to the L1 sequence

Published ChIP-seq data [36] for the four KZFPs that have been reported to bind preferentially to L1Md_Tf were downloaded from GEO (Gm14295, GSM3173710; Gm14406, GSM3173716; Gm14412, GSM3173718; Gm14436, GSM3173722). After trimming using Trim Galore! (−-clip R1 3 --three_prime_clip_R1 1), the reads were mapped onto the mm10 reference genome using Bowtie2. Through deepTools [53], results were then summarized in view of short L1Md_Tf copies (100–400 bp, *n* = 2424) and their flanking regions (10 kb upstream and downstream).

### Analysis of the effect of short L1s in B6 at the genome-wide level

H3K9ac ChIP reads and input reads obtained from the B6 pure strain were counted for each non-overlapping 50-kb bin to identify hyperacetylated regions (fold enrichment of ≥1.5, corresponding to top 3% hyperacetylated regions in B6). L1 copies with the 3′ end that were < 400 bp long were selected (*n* = 845), and ChIP fold enrichment near the sites of L1 insertion (10 kb upstream and downstream) was calculated using deepTools [53].

### Supplementary Information

**Additional file 1: Supplementary Table S1.** List of conserved and strain-specific H3K9ac peaks. **Supplementary Table S2.** List of non-allelic and allele-specific H3K9ac peaks in F1 hybrids. **Supplementary Table S3.** List of LINE indels between B6 and MSM. **Supplementary Table S4.** List of LTR indels between B6 and MSM. **Supplementary Table S5.** Allele frequencies of gene expression in F1 hybrids.

**Additional file 2.**

## Data Availability

The mRNA-seq sequencing data have been deposited to NCBI GEO under the accession number, GSE246401.
