# Supplementary material for: Insertion of short L1 sequences generates inter-strain histone acetylation differences in the mouse
Source: Mob DNA. 2024 May 10;15:11. doi: 10.1186/s13100-024-00321-0 (PMC11084082; doi:10.1186/s13100-024-00321-0)
Supplement: Supplementary file 2 — Additional file 2. [file 13100_2024_321_MOESM2_ESM.pptx]

## Slide 1
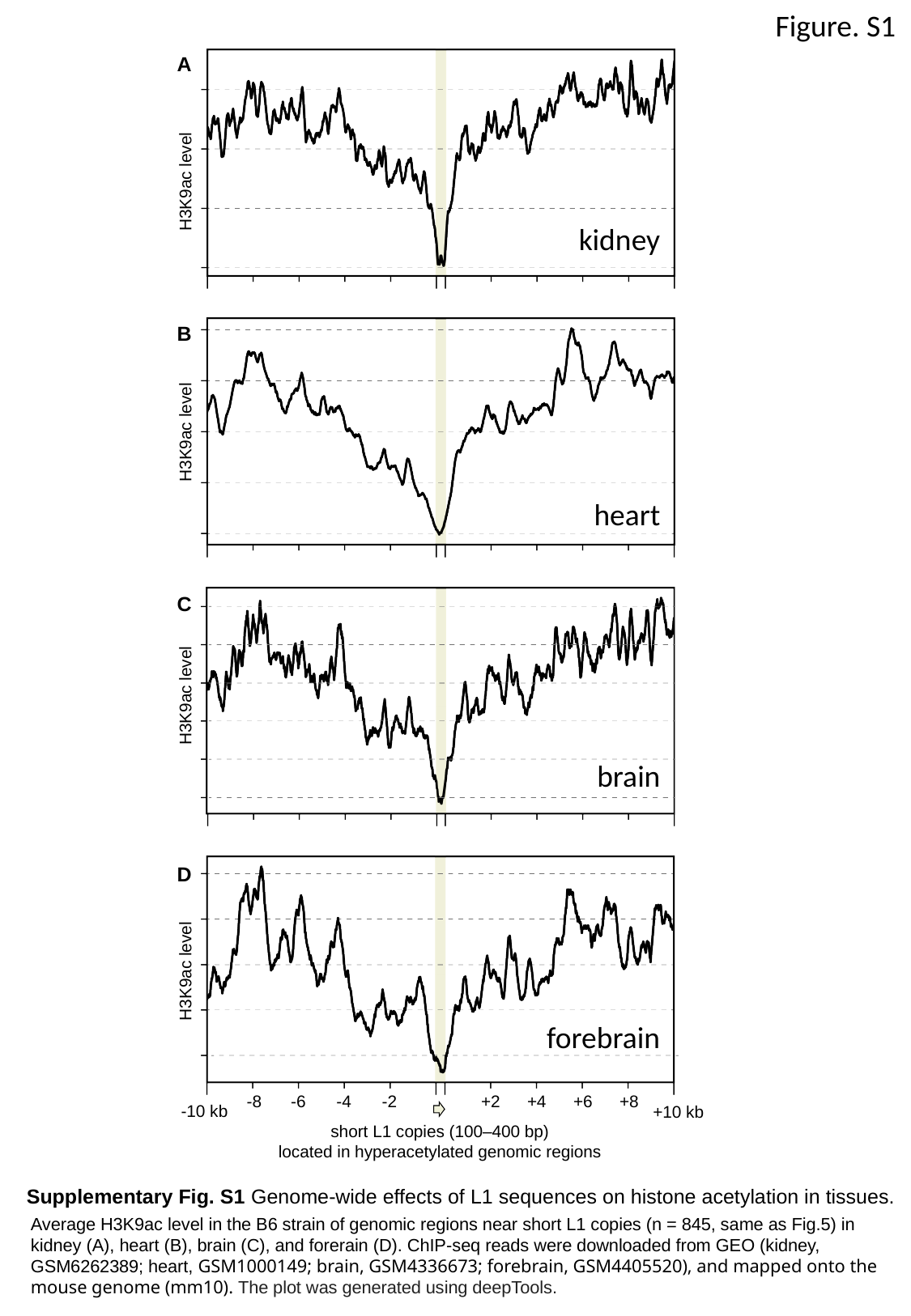

Figure. S1
A
H3K9ac level
kidney
B
H3K9ac level
heart
C
H3K9ac level
brain
D
H3K9ac level
forebrain
-8
-6
-4
-2
+2
+4
+6
+8
-10 kb
+10 kb
short L1 copies (100­–400 bp)
located in hyperacetylated genomic regions
Supplementary Fig. S1 Genome-wide effects of L1 sequences on histone acetylation in tissues.
Average H3K9ac level in the B6 strain of genomic regions near short L1 copies (n = 845, same as Fig.5) in kidney (A), heart (B), brain (C), and forerain (D). ChIP-seq reads were downloaded from GEO (kidney, GSM6262389; heart, GSM1000149; brain, GSM4336673; forebrain, GSM4405520), and mapped onto the mouse genome (mm10). The plot was generated using deepTools.
